# Fecal Myeloperoxidase as a Biomarker for Inflammatory Bowel Disease

**DOI:** 10.7759/cureus.1004

**Published:** 2017-01-31

**Authors:** David R Hansberry, Kush Shah, Prateek Agarwal, Nitin Agarwal

**Affiliations:** 1 Radiology, Thomas Jefferson University Hospitals; 2 Gastroenterology, Rowan University School of Osteopathic Medicine; 3 Neurosurgery, Perelman School of Medicine at the University of Pennsylvania; 4 Department of Neurological Surgery, University of Pittsburgh Medical Center

**Keywords:** myeloperoxidase, biomarker, ulcerative colitis, crohn’s disease, inflammatory bowel disease

## Abstract

Inflammatory bowel disease (IBD) is a chronic condition involving the inflammation of the colon and small intestine. IBD affects as many as 1.4 million people in the U.S. alone and costs the health care industry over $1.7 billion annually. Managing IBD normally requires invasive and often discomforting diagnostic tests. In an effort to alleviate the painful and costly nature of traditional diagnosis, there has been increasing research initiative focused on noninvasive biomarkers. PubMed, provided by the United States National Library of Medicine (NLM) at the National Institutes of Health, was utilized with the following search terms: 1) myeloperoxidase (MPO) 2), inflammatory bowel disease (IBD), and 3) neutrophils. The following terms were used interchangeably with search terms 1-3: 4) costs, 5) biomarkers, 6) review, and 7) etiology. In the context of IBD, myeloperoxidase (MPO), a lysosomal protein found in neutrophils, may serve as a viable biomarker for assessing disease status. Several studies demonstrated increased levels of neutrophils in patients with active IBD. Furthermore, studies have found significantly higher levels of MPO in patients with active IBD compared to patients without IBD as well as patients with inactive IBD. MPO is also expressed in higher concentrations in patients with more severe forms of IBD. When measuring treatment efficacy, MPO levels are indicative of the quality of response. MPO may serve as an important diagnostic and prognostic tool in assessing IBD status.

## Introduction and background

Despite the progress that has been made in biomarker detection technology, challenges remain for improved screening, detection, and treatment of many intractable diseases. In the biomarker research continuum, important steps include the identification of reproducible patterns of disease and response to therapy; capacity to distinguish between health and disease or differentiating diseases when they have overlapping symptoms; collection of specimens, often minute in nature; and platforms that can accurately measure single or multiple markers with rapid, reliable, and reproducible automation technology. All of these challenges are inherent to many diseases, including inflammatory bowel disease (IBD).

Inflammatory bowel disease is a chronic disease of intestinal inflammation that can have serious morbidity and mortality as well as a significant economic burden with annual estimates of total expenditures at $2.8 billion in the United States alone [1]. Frequently, IBD refers to ulcerative colitis (UC) and Crohn’s disease (CD), two of the most prevalent IBDs. Both diseases require extensive diagnostic workup and treatment monitoring, which can be discomforting to the patient. The use of noninvasive diagnostic biomarkers to monitor treatment efficacy and assist in clinical decision-making is beneficial to both the clinician and patient. Many biomarkers have been investigated for clinical usefulness in IBD, including lactoferrin, calprotectin, and myeloperoxidase (MPO). This article reviews the use of MPO as a fecal biomarker in patients with IBD.

PubMed, provided by the United States National Library of Medicine (NLM) at the National Institutes of Health, was utilized with the following search terms: 1) myeloperoxidase (MPO), 2) inflammatory bowel disease (IBD), and 3) neutrophils. The following terms were used interchangeably with search terms 1-3: 4) costs, 5) biomarkers, 6) review, and 7) etiology. References were selected to better understand the role of biomarkers, specifically MPO, in IBD (Table 1) [2-15].

**Table 1:** Selected References on the Role of Fecal Myeloperoxidase (MPO) as a Biomarker for Inflammatory Bowel Disease

Author(s)	Year of Publication	Topic of Interest	Animal Study or Human Study	Sample Size	Sensitivity of MPO	Specificity of MPO	Mean Age
Masoodi, et al. [2]	2012	Ulcerative colitis and Crohn's disease	Human	109	89%	51.4%	38
Saiki T [3]	1998	Ulcerative colitis and Crohn's disease	Human	59	N/A	N/A	32 (median)
Merlie, et al. [4]	1988	Cellular and molecular biology of prostanoid synthesis; isolation and sequence of cDNA of cyclooxygenase	Animal	cDNA library (numerical value not provided)	N/A	N/A	N/A
Kimura and Ikeda Saito [5]	1988	Human MPO and thyroid peroxidase	Human	N/A	N/A	N/A	N/A
Cals, et al. [6]	1991	Bovine lactoperoxidase	Animal	N/A	N/A	N/A	N/A
Klebanoff SJ [7]	2005	Myeloperoxidase activity	Human	N/A	N/A	N/A	N/A
Masoodi, et al. [8]	2011	Ulcerative colitis	Human	N/A	N/A	N/A	N/A
Raab, et al. [9]	1992	Perfusion technique for colorectal perfusion	Human	51	N/A	N/A	N/A
Arnhold J [10]	2004	Properties of MPO	Neither	N/A	N/A	N/A	N/A
Sangfelt, et al. [11]	2001	Biomarkers’ response to local prednisolone treatment in distal ulcerative colitis and proctitis	Human	11	N/A	N/A	45
Peterson, et al. [12]	2002	Quantification of fecal biomarkers, including MPO, in patients with IBD	Human	69	N/A	N/A	43.5 (median)
Lettesjö, et al. [13]	2006	Irritable bowel syndrome (IBS) and collagenous colitis (CC) biomarkers, including MPO	Human	84	N/A	N/A	61 (median)
Wagner, et al. [14]	2008	Evaluation of fecal calprotectin (FC) as a biomarker for IBD and comparison of FC with MPO and fecal eosinophil protein X (EPX)	Human	38	N/A	N/A	40
Sutherland, et al. [15]	2008	Review of a wide range of fecal biomarkers in patients with IBD, including MPO	Human	1,595 (meta-analysis within the article)	N/A	N/A	N/A

## Review

### Myeloperoxidase

Myeloperoxidase is a lysosomal protein that is released into the phagosome of neutrophils during the degranulation process. There, it reacts with hydrogen peroxide and a halide to form hypochlorous acid or with tyrosine to form tyrosyl radicals. These products are highly cytotoxic and can be released from the cell to destroy foreign microorganisms. However, these toxic agents can also damage normal tissue and contribute to inflammation [7]. Myeloperoxidase is often overexpressed in numerous inflammatory diseases, including IBD [16-17]. As a result, MPO has the potential to serve as a viable, noninvasive fecal biomarker for assessing IBD status.

### Neutrophil and IBD studies   

Calprotectin, lactoferrin, and MPO are all associated with inflammation and are derived from neutrophils. Calprotectin is found within the cytosol of neutrophils. Studies have successfully shown elevated levels of calprotectin in patients with IBD and that fecal calprotectin accurately predicts relapses [18]. Lactoferrin is a glycoprotein found in the secondary granules of neutrophils and is resistant to proteolysis in feces [19]. Studies have found it to be both sensitive and accurate in measuring IBD activity [20]. Both calprotectin and lactoferrin are documented biomarkers for monitoring and helping diagnose IBD.

To investigate the relationship between circulating neutrophils and IBD, McCarthy, et al. compared inactivated and activated neutrophils in patients with active CD and UC, inactive CD and UC, and healthy patients. The authors found increased levels of activated circulating neutrophils in active CD and active UC subjects compared to healthy and inactive CD and UC patients [21]. To examine the cellular intricacies behind the characteristic transepithelial neutrophil migration, Kucharzik, et al. studied the tight junction and adherens junction expression levels relative to neutrophil infiltration in patients with IBD. Immunofluorescence data revealed a severe down-regulation of the tight junction protein occludin. The authors concluded that reduced expression of occludin might be involved in the enhanced permeability of neutrophils seen in IBD [22]. To explore the prolonged persistence and accumulation of neutrophils in IBD, Ina, et al. studied the effects of granulocyte colony-stimulating factor (G-CSF) and granulocyte-macrophage CSF (GM-CSF) on neutrophil apoptosis in IBD patients. The authors discovered that patients with active IBD have elevated levels of G-CSF and GM-CSF activity compared to the controls. Furthermore, neutrophils exposed to both factors exhibited concentration-dependent inhibition of apoptosis. The authors suggest that neutrophil apoptosis is delayed due to factors G-CSF and GM-CSF, which may explain why neutrophils accumulate in the tissues of IBD patients [23]. The aforementioned studies describe the relationship between neutrophils and IBD as well as possible mechanisms underlying the nature of neutrophils in IBD.

### MPO and IBD studies

Saiki performed one of the more influential studies concerning the relationship between MPO and IBD. The author measured MPO levels in stool extracts of 33 patients with UC, 32 patients with CD, 9 inflammatory disease control patients, and 15 healthy patients. The author found decreased levels of MPO in the healthy patient population as well as the patients with inactive IBD when compared to patients with active IBD. In fact, there was a statistically significant elevation in MPO levels for active IBD compared to inactive and healthy controls. A paired analysis showed reduced MPO levels after the disease was aggravated and resolved. Saiki further found a linkage between MPO, alternative disease parameters (such as leukocyte counts), and the endoscopic grade of inflammation [3].

Other studies have also been conducted regarding MPO and IBD. Peterson, et al. evaluated different biomarkers in 28 patients with active UC using fecal and serum samples. The authors found elevated fecal MPO levels in all 28 UC patients irrespective of the disease severity. Furthermore, the authors found reduced levels of fecal MPO after four weeks of treatment in 20 of the 28 patients [24]. To further study the role of MPO in assessing IBD activity and response to therapy, Masoodi, et al. examined the fecal MPO levels in 55 patients with UC and 54 age-matched healthy patients. The authors reported elevated MPO levels in the UC patients compared to the control patients. Of the 55 patients with UC, the authors also reported significantly higher fecal MPO levels in the subjects with endoscopically more severe disease compared to those with milder cases of UC [2]. To examine the use of monitoring MPO levels in active IBD, Ping, et al. determined bowel histological scores of 15 active IBD patients, 15 inactive IBD patients, and 12 control patients. The authors determined there was elevated bowel mucosal MPO activity in active and inactive IBD patients compared to control patients. Between patients with active and inactive IBD, bowel mucosal MPO activity was increased in patients with active IBD [25]. These studies suggest and promote measuring MPO levels to assess IBD activity and response to therapy.

### Translational applicability

Several studies have explored the potential use of MPO in clinical practice. Wagner, et al. investigated fecal samples of MPO as an indicator of treatment efficacy for 38 patients with IBD before and after four and eight weeks of treatment. The authors found that increased levels of MPO could indicate an incomplete response to treatment while decreased levels of MPO could indicate a full response to treatment [14]. To examine MPO’s ability to serve as a disease activity marker in IBD, Peterson, et al. measured MPO levels in 44 healthy patients and 18 IBD patients and found relatively higher MPO levels in IBD patients compared to healthy patients. The authors also observed a high discriminative capability for MPO between healthy and IBD patients [12]. These studies argue for MPO as a valuable biomarker for diagnosing and evaluating treatment efficacy of IBD.

There is an obvious need for a noninvasive, reliable, inexpensive, and quick method to assess as well as analyze IBD status and response to treatment in the clinical environment [26]. Currently, the gold standard for monitoring and managing gastrointestinal inflammation is an endoscopy with muscle biopsy. It is supplemented with laboratory tests and radiologic exams [27]. The use of multiple procedures highlights the difficulty in properly checking IBD activity. Recently, the advent of biomarkers has shown promise in resolving such issues. Myeloperoxidase has been studied for IBD diagnostic potential, disease reoccurrence rates, and treatment efficacy [28]. Myeloperoxidase seems to be a prospective biomarker and may serve as a critical tool in sensing and managing IBD. Current costs of detecting and monitoring IBD are approximated to over $500,000 per quality-adjusted life years. These costs are predominately due to performing endoscopy and colonoscopy procedures. Utilizing biomarkers may lower these costs by as much as 31% and have the potential to save patients both time and inconvenience [29]. In fact, a recent study regarding the use of MPO as a biomarker for inflammation in ischemic heart disease suggested that, with new commercial methods, it is feasible to measure MPO at a low cost and high volume [30]. There is a practical application in measuring MPO levels in clinical environments to assess IBD status.

### Discussion

The diagnosis and management of IBD entails lengthy, costly, and invasive procedures. It would be beneficial to monitor IBD using an inexpensive, consistent, and rapid test. The realm of biomarkers provides a promising avenue to reliably monitor IBD activity. The logic behind fecal biomarkers is that they are in direct contact with the intestine and should contain markers specific to IBD. The presence of these biomarkers indicate the onset and severity of IBD [15]. One characteristic feature of IBD is the infiltration of neutrophils into the gastrointestinal tract wall as well as expulsion of neutrophil proteins into the bowel lumen. With this in mind, MPO, a lysosomal protein found within neutrophils, is thought to be a potential fecal biomarker in evaluating IBD. Several preliminary studies have explored and validate its use as a fecal biomarker in assessing bowel disease activity. Both Masoodi, et al. and Ping, et al. found elevated MPO levels in patients with IBD compared to healthy patients [2, 25]. Furthermore, their studies revealed that fecal MPO levels are higher in patients with more severe forms of IBD. Wagner, et al. and Peterson, et al. examined the relationship between fecal MPO levels and IBD treatment [12, 14]. These authors showed reduced levels of fecal MPO after treatment and that fecal MPO levels indicate the degree of responsiveness to IBD treatment. 

## Conclusions

The future of MPO in evaluating IBD is promising. Not only does it hold promise to be cost-effective to measure MPO levels to assess IBD status, it is also safer and more convenient for the patient as well as the clinician. The possibility of utilizing a biomarker like MPO is rational given its involvement in IBD. Further large population studies need to be done to better understand and validate MPO's diagnostic and prognostic value in assessing IBD. However, due to its specificity, simplicity, non-invasive, and likely inexpensive nature, MPO may serve as an important tool for evaluating patients with IBD.
